# Impacts of seasonal variations and wastewater discharge on river quality and associated human health risks: A case of northwest Dhaka, Bangladesh

**DOI:** 10.1016/j.heliyon.2023.e18171

**Published:** 2023-07-11

**Authors:** Hazzaz Bin Hassan, Md. Moniruzzaman, Ratan Kumar Majumder, Fowzia Ahmed, Md. Abdul Quaiyum Bhuiyan, Md. Ariful Ahsan, Hafiz Al-Asad

**Affiliations:** a Department of Environmental Science, Bangladesh University of Professionals (BUP), Mirpur-12, Cantonment, Dhaka, 1216, Bangladesh; b Isotope Hydrology Division, Institute of Nuclear Science and Technology, Atomic Energy Research Establishment, Bangladesh Atomic Energy Commission, Savar, Dhaka 1349, Bangladesh; c Department of Chemistry, Mawlana Bhashani Science and Technology University, Santosh, Tangail, 1902, Bangladesh

**Keywords:** Effluent, CETP, EWQI, Carcinogenic, Bioaccumulation, Biodegradability

## Abstract

Surface water pollution caused by the discharge of effluents from industrial estates has become a major concern for Dhaka (Bangladesh). This study aims to have a concise look at the severe river water pollution, mainly from effluents discharged from the tannery village. Effluent samples were collected from five ejected points, including the central effluent treatment plant (CETP), twenty adjacent river water, and two pond water nearby Hemayetpur, Savar. Thirty-one parameters have been observed at these sampling points for three seasons, from April 2021 to January 2022. The results obtained from water quality indices, i.e., water quality index (WQI), entropy water quality index (EWQI), and irrigation water quality index (IWQI), show that most studied surface water samples ranked “unsuitable” for consumption, irrigation, and anthropogenic purposes. The highest health risk was observed downstream of Hemayetpur city at the Savar CETP discharge site, indicating higher levels of heavy metal in the river water following the tannery village. Carcinogenic and non-carcinogenic human health risks could be triggered mainly by water consumption as concentrations of arsenic (As), chromium (Cr), nickel (Ni), and lead (Pb) exceeded the upper benchmark of 1 × 10^−4^ for adults and children. The results of the carcinogenic risk assessment revealed that children were more vulnerable to health hazards, and quick corrective action is required to control the increased levels of heavy metals at all sample locations. Therefore, through bioaccumulation, human health and the environment are affected in these areas. Using river water for consumption, household work, or even irrigation purposes is not advisable. This study's result highlighted that properly implementing compatible policies and programs is required to improve effluent treatment methods and provide biodegradability to the Dhaleshwari River.

## Highlights

• Water quality of Dhaleshwari river is examined around Savar Tannery Estate.
• The maximum values of Cr, Ni, Pb, Cd and Mn exceed all permissible limit.
• Degradation of water quality is significant due to improper effluent discharged.
• Health risk to human is very high through consumption of contaminated water.

## Graphical abstract

**Figure:**
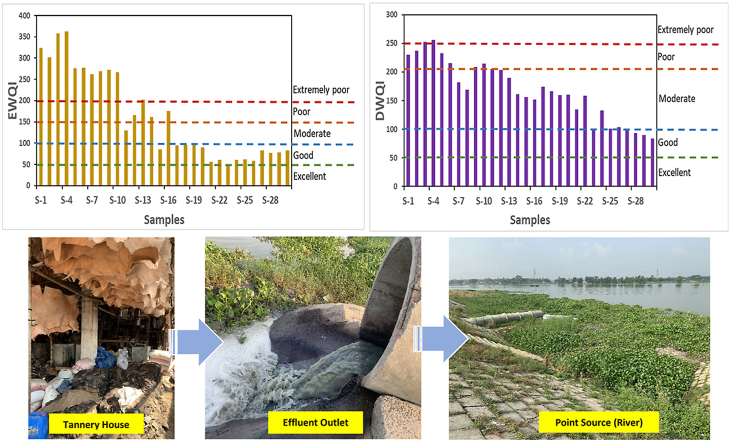

## 1 Introduction

Water management and water quality are essential for the ecological environment and urban economy, particularly in areas with severe water shortages. Recently, with the increase of massive populations, countries like China, Turkey, Kenya, Bangladesh, and others have been facing severe water scarcity problems due to modern industrialization [[1], [2], [3], [4], [5], [6]]. Surface water is particularly susceptible to contamination due to anthropogenic factors such as industrial, agricultural, and waste discharge, as well as the convenience of treating wastewater [7,8].

Bangladesh has water distributaries with rivers, lakes, ponds, haors, and beels. The industry like tanneries is always keen to have rivers face due to consumption of water required for production. Discharging untreated effluents from tanneries' physical, chemical, and biological properties deteriorates the receiving water bodies' [[9], [10], [11], [12], [13]]. Streams adjacent to the industries are affected mainly by effluents containing a high number of heavy metals. Industries are responsible for annually discharging 300–400 million tons of heavy metals, solvents, toxic sludge, and other pollutants into the water around the world [[14], [15], [16], [17]]. Refining 1 kg of skin required 40 L of water, which resulted in the production of a large amount of effluent, leading to an increase in physiochemical parameters [[18], [19], [20]].

Before moving to Savar, the leather industry estate in Hajaribagh produced hundreds of metric tons of organic and inorganic waste, which contaminated the adjacent streams, including the Buriganga River [21,22]. Every year, 60,000 tons of unprocessed raw hides and skins were treated in Hajaribagh tanneries, but not in the most effective methods. The leather industry of Bangladesh is categorized and enlisted as a “red industry” by the Bangladesh Environmental Conservation Act (ECA, 1995) and environmental conservation rules (ECR, 1997) [23]. The way toward migrating tanneries from Hazaribagh to the tannery village of Savar near the Dhaleshwari river, was finished in 2017. According to the Bangladesh Tanners Association (BTA), about 156 factories have started working, and a few remain under construction. The balance of the aquatic system of the adjacent river is at risk due to anthropogenic influences. Although the water is almost appropriate for irrigation and in good condition for the aquatic biota, adequate measures are still needed to treat discharged effluents [24,25].

CETP has been set up to treat the significant effluent discharge from the existing industries, yet it is not completely functioning. These could affect the nearby Dhaleshwari river without proper treatment, as the heavy metals released with the effluents may have a high contamination rate [26]. To terminate the pollution from small-scale cluster tanneries, the aim is to form a CETP made up of reinforced concrete structures [27]. Sodium sulfate, ammonium nitrate, sodium chloride, and sodium sulfide chemicals are used for tanning, which are not mixed entirely. The remaining chemicals mixed with the effluent that can come into contact with the CETP surface may lead to degradation of the concrete as well as river water, which has been a growing environmental issue [12,28]. For now, the precise effects of these contaminants on water quality are unknown.

Water quality indices are tools for determining water quality requirements and comparable to any other method, require understanding of the fundamental concepts of water and related issues [29]. However, much attention has been given in recent years to the evaluation of water quality indices in ground and surface water, with the development of an entropy-based water quality index (EWQI). Numerous heavy metal pollution indices have been assessed during last decades [30,31]. To assess the different presence of heavy metals in effluent and river water, a human health risk assessment was investigated using ingestion and dermal pathways for population. Surface water quality parameters are essential in evaluating the eligibility of water for irrigation. Water quality for crop irrigation tends to involve several issues, as well as water quantity and availability.

This study applied different water quality indices and health risk assessments to assess the water quality and spatial variations in the Dhaleshwari river. This study aims to evaluate the physicochemical parameters of river water and effluents as well as the heavy metals discharged from the CETP to the Dhaleshwari River and their impact on human health. The current study aims to 1) evaluate the likely sources of toxic metal (oid)s and their diffusion in river water; 2) determine the seasonal variations in contamination level using a statistical approach; 3) examine the carcinogenic and non-carcinogenic hazard of metal (oid)s via ingestion and dermal exposure routes of surface water; and 4) contribute to the data-record for long term sustainable and environmental initiatives in addition to effective public policy implementation for effluent discharge. Hence, this research will be the pioneer in bringing out the human health risks for the region.

## 2 Materials and methods

### 2.1 Study area

**Fig. 1:**
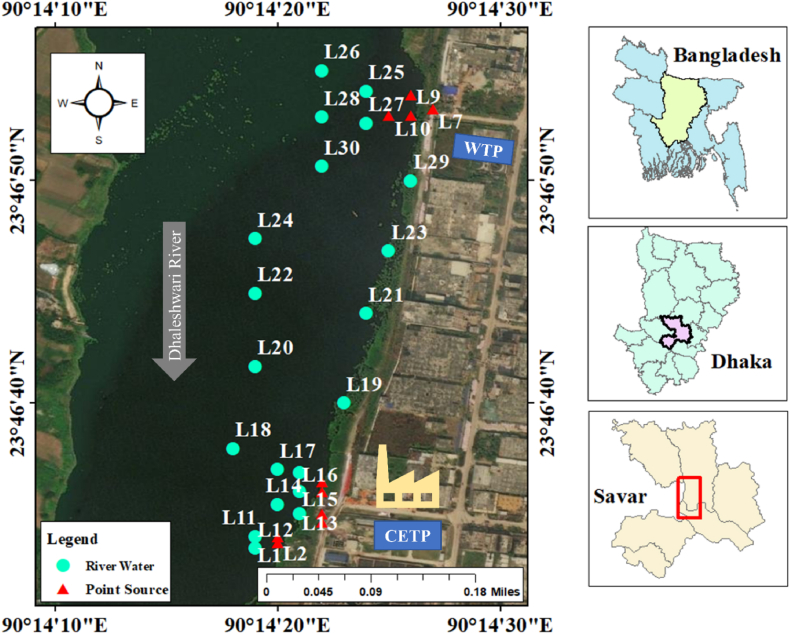
Maps showing the physiography and the sampling locations around Central Effluent Treatment Plant (CETP) and Water Treatment Plant (WTP) in the Savar district, Dhaka.

The study was carried out on Tannery Industrial Estate, which is located in the Hemayetpur region at Savar, Dhaka. The total length of the Tannery Estate is approximately 2 km, with CETP, and is situated adjacent to the Dhaleshwari River. The total length of the Tannery Estate is about 2.5 km, and the effluent discharges to the downstream border of the Dhaleshwari River from CETP. Two different climatic types, tropical wet and dry, are observed in this region. Most of the region usually has moderate precipitation throughout the year. The average precipitation of the region is 74.11 mm annually [32,33]. This geographical area's predominant land use patterns are depressions, agricultural lands, and settlements [34,35]. Treated and untreated effluents are directly discharged from the industrial estate into the river. Hence, the effluent properties discharged from the treatment plant and hydrologic conditions were investigated. Thus, a location map of sampling points was prepared using ArcGIS software based on the data observed (Fig. 1).

### 2.2 Sample collection and analysis

Three sampling stations were carefully selected to represent the discharged effluents and flow to the river, considering the locations' criteria. In the present study, sampling points were selected, including surface river water and points at both stream ends of the Dhaleshwari river. Additionally, samples were explicitly collected from the sites representing punctual and diffuse sources of pollutants in the surface water. This study was performed based on treated tannery effluent samples (n = 10) and adjacent surface water samples (n = 20) near CETP, in Savar, Bangladesh. The samples were collected during the pre-monsoon, post-monsoon, and winter periods. Due to early precipitation in the monsoon season, surface water flow has substantially increased during this period. Representative water samples were collected to avoid the presence of air fluctuations. Each sampling location point was taken with a handheld GPS (Global Positioning System) and then plotted on a map (Fig. 1). Standard procedures are followed as recommended by the American Public Health Association (APHA) [36]. Samples were collected for cation, anion, and trace element analysis. Each bottle was kept unacidified for anion analyses. The other bottle was acidified to pH < 2 b y adding several drops of ultra-pure nitric acid in plastic bottles (500 mL) for determining cations and preserved in the laboratory at 4 C in the refrigerator.

Water temperature, pH, redox potential (Eh), dissolved oxygen (DO), electrical conductivity (EC), and total dissolved solids (TDS) were measured in situ at each sampling location using *HACH* multiparameters subjected to a complete analysis of physicochemical parameters. Bicarbonate (HCO_3_^−^) was measured following acid titration (endpoint method). Samples for chemical analysis of different inorganic constituents (anions and cations) and trace elements were immediately filtered through 0.22 μm cellulose membranes. The major anions were analyzed using an ion chromatograph (Model: *Dionex ICS 3000*), while the cations were by AAS-Atomic Absorption Spectrophotometer (Model: *ZEEnit 700*); Sodium (Na^+^) and Potassium (K^+^) were analyzed using a flame photometer (Model: *PFP7 & PFP7/C*). For trace metal analysis, all samples were acid digested (65% HNO_3_) and concentrated by heating gently on the hot plate at 95–105 °C. The samples prepared for the study were filtered using ashless 42 sizes (pore size 2.5 μm) Whatman filter paper. The concentrations of 12 metals (Fe, Cd, Hg, As, Al, Zn, Ni, Pb, Co, Mn, Cu and Cr) were determined using Atomic Absorption Spectrophotometer (AAS) (ZEEnit 700). The precisions of chemical analysis were carefully examined by taking and analyzing blanks and replicate samples, and accuracy by five standard samples, respectively. The concentration found in AAS was interpreted as mg/L for water samples.

Water samples collected for the study were analyzed at the Isotope Hydrology Division, Institute of Nuclear Science and Technology (INST), Bangladesh Atomic Energy Commission (BAEC), Savar, Dhaka, Bangladesh.

### 2.3 Water quality indices

Numerous approaches have been established to evaluate the water chemistry and condition of water quality in the river [37,38]. Water quality indices are tools for determining water quality requirements, and comparable to any other method, they require an understanding of the fundamental concepts of water and related issues [37,[39], [40], [41]]. The primary goal of any water quality monitoring study is to assess the water quality status for special uses. Water quality indices have been developed around the world, such as Entropy Water Quality Index (EWQI) and the Canadian Council of Ministers of the Environment Water Quality Index (CCMEWQI) [42,43]. However, much attention has been given in recent years to the evaluation of water quality indices in ground and surface water, with the development of the entropy-based water quality index (EWQI) [[44], [45], [46], [47]]. This method seamlessly integrates numerous environmental parameters into a single value indicating the state of water quality. As a result, rather than comparing the different assessment results of multiple variables, the EWQI method is an effective water quality assessment technique that offers comprehensive information about overall quality. To represent the quality of the water, ranges have been introduced as excellent, suitable, medium, poor, and extremely poor.

#### 2.3.1 Entropy water quality index (EWQI)

The entropy principle was initiated by C. Shannon [48], who anticipated the result of a probabilistic occurrence by demonstrating the degree of uncertainty [49,50,98,99]. This index offers a neutral assessment of water quality taking into account all the parameters assessed for each water sample [46]. Parameters evaluated in this study include anions, cations, and heavy metals (i.e., Fe, Mn, As, Cr, Ni, Cu, Zn, Pb).

The computation of the equation matrix (C) is the initial step, which relates to water samples (m) and parameters (n). The equation matrix (C) is computed by using Equation (1):

(1) $$C=\left[\begin{array}{cccc} C_{11} & C_{12} & \cdots & C_{1n}\\ C_{21} & C_{22} & \cdots & C_{2n}\\ \vdots & \vdots & \ddots & \vdots \\ C_{m1} & C_{m2} & \cdots & C_{mn} \end{array}\right]$$

Afterward, the equation matrix (C) is converted into a conventional matrix (R) to minimize the effect of various units and quantity grades of water quality variables; conventional matrix (R) can be computed from the transformed equation matrix (C) by Eq. (2):

(2) $$R=\left[\begin{array}{cccc} R_{11} & R_{12} & \cdots & R_{1n}\\ R_{21} & R_{22} & \cdots & R_{2n}\\ \vdots & \vdots & \ddots & \vdots \\ R_{m1} & R_{m2} & \cdots & R_{mn} \end{array}\right]$$

The ratio of the parameter index value j in sample i is then calculated from Eq. (3):

(3) $$P_{ij=}R_{ij}/\sum_{i=1}^{m}C_{ij}$$

And therefore, the information entropy ($e_{j}$) can be calculated from Eq. (4):

(4) $$e_{j}=-\frac{1}{\ln\;m}\;\sum_{i=1}^{m}P_{ij\;\ln\;P_{ij}}$$

The greater effective implementation of parameter j is dependent on the entropy level. Finally, the entropy weight ($\omega_{j}$) for j parameter is quantified using the information entropy ($e_{j}$) in Eq. (5):

(5) $$\omega_{j}=\frac{1-e_{j}}{\sum_{j=1}^{n}1-e_{j}}$$

The second step of EWQI estimation is the identification of the qualitative rating scale ($q_{j}$) for each variable by Eq. (6):

(6) $$q_{j}=\frac{C_{j}}{S_{j}}\times 100$$

where $C_{j}$ is the parameter concentration for the ions and $S_{j}$ is the parameter concentration for the allowable surface water quality recommendations (mg/L). E.C.R. ‘97, United States Environmental Protection Agency (USEPA) and World Health Organization (WHO) has been used for the standards.

Lastly, the final step of EWQI can be calculated from Eq. (7):

(7) $$EWQI=\sum_{j=1}^{n}\omega_{j}q_{j}$$

According to Unigwe [49], the EWQI for anthropogenic uses can be divided into five categories, ranging from “Excellent” to “Unfit for human consumption or Undrinkable” water (Table 2).

**Table 1:** Summary data of water quality parameters concentrations detected in effluents (n = 10) and river water (n = 20) samples from the Industrial Estate on Hemayetpur, Dhaka.

	Pre-Monsoon			Post-Monsoon			Winter			Bangladesh National Standard
	PS	RS	MR	PS	RS	MR	PS	RS	MR	
Temperature	31.26	28.12	28.23	31.26	28.12	28.23	31.26	28.12	28.23	–
pH	10.24	8.06	7.91	10.15	7.97	7.82	10.71	8.53	8.38	6–9
Eh	−232.09	−110.07	−99.03	−232.09	−110.07	−99.03	−232.09	−110.07	−99.03	–
EC	8624.80	2930.90	2967.20	6879.80	2117.50	2106.00	8482.80	2788.90	2825.20	1500
DO	0.58	4.63	4.22	0.66	4.71	4.30	0.50	4.55	4.14	4.5–8
TDS (mg/L)	4757.80	1528.30	776.30	3534.80	934.10	391.70	4435.80	1317.10	620.50	2100
Salinity	4.83	1.57	0.87	3.13	0.89	0.87	4.43	1.35	0.77	–
Fluoride (mg/L)	69.92	40.53	44.87	47.40	25.86	30.20	76.74	55.20	59.54	7
Chloride (mg/L)	6399.41	793.94	349.75	5246.75	636.50	192.31	6053.97	617.30	332.31	600
Nitrite (mg/L)	214.42	16.76	10.88	224.42	26.76	10.88	214.42	16.76	10.88	45
Nitrate (mg/L)	408.19	371.79	147.09	272.86	236.45	66.68	284.73	304.28	116.88	45
Sulfate (mg/L)	4099.13	1145.78	473.15	2353.91	598.48	297.70	3753.38	935.35	398.03	400
Phosphate (mg/L)	42.61	65.78	30.81	33.99	57.16	22.19	38.06	61.24	26.26	6
Alkalinity (mg/L)	614.70	170.40	95.60	539.70	145.00	82.60	576.70	163.40	88.00	600
Sodium (mg/L)	254.92	158.87	162.94	179.74	83.70	87.76	216.15	120.11	124.17	200
Potassium (mg/L)	81.05	55.89	60.47	68.60	43.44	48.02	73.48	48.33	52.91	12
Calcium (mg/L)	22.08	7.79	11.74	20.30	6.02	9.97	19.96	5.67	9.63	75
Magnesium (mg/L)	42.32	7.67	4.59	35.30	5.29	3.36	45.34	10.69	7.61	35
Silicon Dioxide (mg/L)	847.03	500.00	348.44	729.81	382.78	231.22	769.58	422.55	270.99	–

N·B: PS= Point Source, RS= River Side, MR = Mid River.

**Table 2:** Categorical differences between EWQI, CCME WQI and DWQI.

Index Method	Water Quality	% of Sample		References
		Effluent Sites (10)	River Water (20)	
**EWQI**	Excellent (<25)	–	–	[45,49]
	Good (25–50)	–	–	
	Medium (50–100)	–	75	
	Poor (100–150)	–	5	
	Extremely poor (>150)	100	20	
**CCME WQI**	Excellent (95–100)	–	–	[34,41]
	Good (80–94)	–	–	
	Fair (65–79)	–	–	
	Marginal (45–64)	–	–	
	Poor (0–44)	100	100	
**DWQI**	Excellent (0–25)	–	–	[37,92]
	Good (26–50)	–	–	
	Poor (51–75)	–	–	
	Very Poor (76–100)	–	25	
	Unsuitable (>100)	100	75	

#### 2.3.2 Canadian Council of Ministers of the environment water quality index (CCMEWQI)

The Canadian Council of Ministers of the Environment established this water quality index using a formula that the British Columbia Ministry of Environment provided. Since the water quality characteristics vary from location to location and rely on environmental circumstances, CCME-WQI does not categorize any water quality parameters or periods. The calculation of this index requires a minimum of four parameters and a minimum of four measurements of each of these variables [[51], [52], [53]]. The CCME included an aggregation function that is very different from that of other models. equations (8), (9), (10), (11), (12), (13) stated as:

(8) $$WQI=100-\frac{\surd F_{1}^{2}+F_{2}^{2}+F_{2}^{2}}{1.732}$$

(9) $$F_{1}=\frac{no.\;of\;failed\;variables}{total\;no.\;of\;variables}\times 100$$

(10) $$F_{2}=\frac{no.\;of\;failed\;tests}{total\;no.\;of\;tests}\times 100$$

(11) $$F_{3}=\frac{nse}{0.01nse+0.01}$$

(12) $$nse=\frac{\sum excursion}{total\;number\;of\;tests}$$

(13) $$excursion=\frac{failed\;tests\;test}{standard\;value}-1$$

Ranked indicators illustrate [Poor (0−44), Marginal (45–64), Fair (65–79), Good (80–94), and Excellent (95–100)] the condition of water quality.

### 2.4 Irrigation water quality indices

Surface water quality parameters are essential in evaluating the eligibility of water for irrigation [54]. Water quality for crop irrigation involves several issues, including water quantity and availability. Moreover, quality aspects are ignored while considering surface water quantity. EC, TDS, major cations, and anions are commonly used to define irrigation water quality [55]. Around the world, the three most common problems raised for surface water quality are salinity, specific ion toxicity, and reduced permeability. The irrigation water quality indicators have been computed utilizing several criteria [56]. To determine the surface water quality parameters such as Total Hardness (TH), Sodium Percentage (Na%), Sodium Adsorption Ratio (SAR), Soluble Sodium Percentage (SSP), Residual Sodium Bicarbonate (RSBC), Permeability Index (PI), Magnesium Adsorption Ratio (MAR) and Kelley's Ratio (KR) were assessed for the suitability of irrigation.

#### 2.4.1 Total hardness

The following equation (14) is used to calculate total hardness (TH) in mg/L [57,58]:

(14) $$TH=2.497{Ca}^{2+}+4.11{Mg}^{2+}$$

#### 2.4.2 Sodium percentage (na %)

The sodium content of irrigation water is commonly expressed as Na%. The parameters were determined using water samples' chemical variability [59]. Eq. (15) used to calculate the Na % as:

(15) $$Na\%=\frac{{Na}^{+}}{{Ca}^{2+}+{Mg}^{2+}+{Na}^{+}}\times 100$$

#### 2.4.3 Sodim adsorption ratio (SAR)

Richards [60] first proposed this parameter, which is used to assess the tendency of Na ions to adsorb on soil. The US Salinity Laboratory expresses the Sodium adsorption ratio (SAR) [61], Eq. (16) stated SAR as:

(16) $$SAR=\frac{{Na}^{+}+\sqrt{{HCO}_{3}^{-}}}{\sqrt{{(Ca}^{2+}+{Mg}^{2+})/2}}$$

#### 2.4.4 Soluble sodium percentage (SSP)

Eq. (17) expressed soluble sodium percentage (SSP) as:

(17) $$SSP=\frac{{Na}^{+}+K^{+}}{{Ca}^{2+}+{Mg}^{2+}+{Na}^{+}+K^{+}}\times 100$$

#### 2.4.5 Residual sodium bicarbonate (RSBC)

The residual sodium bicarbonate (RSBC) was adopted to determine irrigation water quality [62]. RSBC computed as Eq. (18):

(18) $$RSBC={HCO}_{3}^{-}-{Ca}^{2+}$$

#### 2.4.6 Permeability index (PI)

The surface water permeability was evaluated depending on the water quality by calculating the permeability index (PI) using the below equation (19). If PI <75%, the water is suitable for irrigation, but if PI >75%, water is not permissible for irrigation purposes [54].

Permeability index (PI) is defined as:

(19) $$PI=\frac{{Na}^{+}+\sqrt{{HCO}_{3}^{-}}}{{Ca}^{2+}+{Mg}^{2+}+{Na}^{+}}\times 100$$

#### 2.4.7 Magnesium adsorption ratio (MAR)

The Magnesium adsorption ratio (MAR) was used to ensure irrigation water adequacy [63]. MAR, also known as magnesium hazard (MH), was computed as Eq. (20):

(20) $$MAR=\frac{{Mg}^{+}}{{Ca}^{2+}+{Mg}^{2+}}\times 100$$

#### 2.4.8 Kelley's ratio (KR)

The Kelly ratio (KR) is another vital parameter for assessing irrigation surface water quality [64]. It was calculated by comparing the concentration of Na ions to the rates of Ca and Mg ions. KR > 1 denotes increased Na in the water [65]. And at last, Kelly's Ratio (KR) is represented in Eq. (21) as:

(21) $$KR=\frac{{Na}^{+}}{{Ca}^{2+}+{Mg}^{2+}}$$

All ionic concentrations have been expressed in milliequivalents per liter (meq/L). To analyze the surface water's suitability for irrigation water, each of the parameters and individual chemical parameters were compared to E.C.R. ‘97, the United States Environmental Protection Agency (USEPA), and the World Health Organization (WHO).

### 2.5 Human health risk assessment

Metal(oid)s such as manganese (Mn), nickel (Ni), chromium (Cr), copper (Co), cadmium (Cd), lead (Pb), zinc (Zn), aluminum (Al), arsenic (As), and mercury (Hg) are commonly found in effluents of tannery industries and classified as “potential human health risks” via consumption [66,67]. Numerous heavy metal pollution indices have been assessed during the last few decades [68]. To evaluate the types of HMs in the effluent and river water, a human health risk assessment examination using ingestion and dermal pathways was conducted for the population. The health risk assessment was carried out following the approaches recommended by the USEPA [69,70]. This method is widely used to assess heavy metal exposure in water concerning human health. Metal concentrations (Fe, Mn, As, NO_3_-, Cr, Co, Ni, Cu, Zn and Pb) were measured and used to evaluate the health consequences for adults and children. For evaluating health risks caused by trace elements in water, direct oral ingestion and dermal absorption by the skin are generally considered [[71], [72], [73]].

(22) $${ADD}_{Oral}=\frac{\left(CW\times IR\times EF\times ED\right)}{BW\times AT}$$

(23) $${ADD}_{Dermal}=\frac{\left(CW\times SA\times K_{P}\times IR\times ET\times EF\times ED\times CF\right)}{BW\times AT}$$

Where, ADD = Average daily dose intakeCW = Concentration of metal presence in water (mg/L)IR = Ingestion rate (L/day)EF = Exposure frequency (days/year)ED = Exposure duration (year)BW = Body weight of the exposed individual (kg)AT = Time period over which the dose is averaged (days)ET = Exposure time (h/day).

In equations (22), (23)) mentioned above, and denotes the dose of exposure (mg/kg/day) via oral intake and dermal pathway, calculated using the parameters values of USEPA [70]. To assess the non-carcinogenic health risk of contaminant exposure, the hazard quotient (HQ) was calculated for both the oral and dermal pathways [69].

(24) $$HQ=\frac{ADD}{RfD}$$

ADD is the average daily dose intake, and RfD indicates the reference dose of calculated contaminant (mg/kg/day), taken into account from the risk-based concentration table [74]. Population exposed to HQ > 1 has an undesirable risk of an adverse non-carcinogenic effect on human health [75].

(25) $$HI={HQ}_{i}+{HQ}_{ii}+{HQ}_{iii}\ldots \ldots \ldots \ldots +{HQ}_{n}$$

(26) $$HI=\sum_{i=1}^{2}{HQ}_{i}$$

Hazard Index (HI) represents the sum of total HQs as mentioned in Eq. (25), (26)). While ΣHQ is ≤ 1, the metal has no potential risk in the indexing method [76,77].

Carcinogenic risk is the probability of acquiring any kind of cancer after prolonged exposure to carcinogenic exposures. The acceptable or tolerable risk threshold ranges from 10^−6^ to 10^−4^ [78].

(27) CR=ADD×CSF

Carcinogenic risk was evaluated for As, Cr and Pb; CSF values are 1.5, 0.5 and 0.0085 (mg/kg/day)^−1^ for oral intake and 3.66, 20, 42.5 (mg/kg/day)^−1^ for dermal respectively [44,79].

### 2.6 GIS and statistical analyses

ArcGIS version 10.7's Inverse distance weighted (IDW) interpolation method was used to project the spatial distribution of WQI scores and the research area map. Excel and SPSS 20.0 were used for the statistical analysis of the data.

## 3 Results and discussions

### 3.1 Difference in physicochemical characteristics between treated effluent sites and river water

Table 1 displays the results of the investigation carried out to determine the spatial and temporal physicochemical characteristics of the Dhaleshwari River surface water. In addition, Table 1 provides descriptive statistics for the water quality parameters. The current study assessed the status of anions, cations, and heavy metals in river water at different seasons to evaluate pollution levels. The mean pH values of the effluent sites and river water were measured at 10.24 and 8.06. These results show that water samples from effluent sites have greater alkaline properties than river water. A high pH was determined in the areas where effluents were discharged from the industrial zones. Increased levels of pH tend to have significant effects on environments.

Additionally, effluent water samples taken from the discharge pipeline were measured in pre-monsoon (4757 mg/L), post-monsoon (3534 mg/L) and winter (4435 mg/L) have high Total Dissolved Solids (TDS) compared to the river water in pre-monsoon (1528 mg/L), post-monsoon (934 mg/L) and winter (1317 mg/L), respectively. Apart from having an unfavorable effect on the water's other qualities, TDS has no direct impact on human health. Water samples' Electrical Conductivity (EC) varies premonsoon (8624 μs/cm), postmonsoon (6879 μs/cm) and winter (8482 μs/cm) in effluent sites, and premonsoon (2930 μs/cm), postmonsoon (2117 μs/cm) and winter (2788 μs/cm) in river water. Pollutants from the industrial sites increase the effluents' ion contents, resulting in maximum EC values. The amount of solids dissolved in water is directly proportional to the electrical conductivity of the water [37]. The water samples temperatures varied from 28 to 31 °C in the post-monsoon period between the effluent site and river water. An extensive range of variation is observed in the water samples' pH, TDS, EC, and temperature as the wastewater is discharged into surface water. In addition, the difference may also be due to agricultural runoff and household activities. Chloride concentrations in the present study ranged from 6399 mg/L in premonsoon, 5246 mg/L in postmonsoon and 6053 mg/L in winter; 793 mg/L in premonsoon, 636 mg/L in postmonsoon and mg/L 617 in winter in effluent sites and river water. Substantial Cl^−^ concentrations in the premonsoon and winter, suggest the hydrologic influence and wastewater contamination in the river water (Table 1). The excessive chloride ions present in the water samples is due to sodium chloride, which is used in the pickling process, i.e., hide and skin preservation. Sulfate concentrations went from 4099 mg/L, 2353 mg/L, and 3753 mg/L in the effluent sites; 1145 mg/L, 598 mg/L and 935 mg/L in the river water, concerning the seasonal variations for the three seasons. The primary source of sulfate is sodium sulfate in chrome tanning powder. All the values of chloride and sulfate are very high compared to the standard limit recommended by ECR ‘97 and inland water guidelines for discharging effluents into surface water, indicating a toxic environment for the inland water biota. Numerous tanning projects in the industrial zone are noteworthy for the high salt content in the effluent and river water [80].

Nitrite and nitrate concentrations in water samples were 214 mg/L and 408 mg/L in the pre-monsoon, 224 mg/L and 272 mg/L in the post-monsoon, and 214 mg/L and 284 mg/L in the winter at the effluent sites. On the other hand, river water nitrite and nitrate concentrations were 16 mg/L and 371 mg/L in pre-monsoon, 26 mg/L and 236 mg/L in post-monsoon, and 16 mg/L and 304 mg/L in winter, respectively. Several auxiliary components of nitrogen, such as ammonia, are used for liming/unhearing operations increase of N_2_ in tannery effluents leads to eutrophication of the adjacent water body [12]. Consumption of water contaminated with nitrates may cause severe diseases in humans and infants, i.e., blue babies or methemoglobinemia, gastric carcinomas, abnormal pain and diabetes [81,82,97]. Alkalinity concentrations vary from 614 mg/L to 170 mg/L, 539 mg/L to 145 mg/L, and 576 mg/L to 163 mg/L in effluent sites and river water for the three seasons, respectively (Table 1). Increases in anion contents were observed in the effluent sites at the industrial zone. In contrast, decreases have been observed with the distance from the effluent outlet to downstream river water.

According to the study, the sodium concentration in effluent locations and river water channels varied from 254 mg/L to 158 mg/L; 179 mg/L to 83 mg/L and 216 mg/L to 120 mg/L in pre-mosoon, post-monsoon, and winter, respectively (Table 1). The overuse of Na salts caused elevated Na concentrations during the tanning process. Similarly, magnesium (Mg) levels in tannery effluents may have arisen due to the use of Mg compounds in basification [83]. Potassium, calcium, and silicon constituents in effluent sites were 81 mg/L, 22 mg/L, and 847 mg/L in pre-monsoon, 68 mg/L, 20 mg/L, and 729 mg/L in post-monsoon, and 73 mg/L, 19 mg/L, and 769 mg/L in winter, respectively. When comparing the effluent site analysis with river water, higher concentrations were measured in the effluent sites for all major ions. Regarding the physical and chemical parameters of all the samples analyzed, the worst level was found at effluent sites, which indicates the presence of waste from the leather industry. Flow conditions of river water, seasonal influences, and human impacts were linked to large regional and temporal changes of these parameters. During the monsoon season, the significant amount of precipitation may have substantially diluted the stream, leading to a considerable improvement in water quality, whereas during the dry season, the reverse have occurred.

The result (Table 1) showed that all the parameters, including anions and cations, exceeded the standard derived for surface water quality (except iron). The concentration of measured parameters in effluent sites followed a decreasing order of: Anions: Cl^−^ > SO_4_^2-^ > NO_2_^−^ > HCO_3_^−^ > NO_3_^−^ > F^−^ > PO_4_^3−^; Cations: SiO_2_ > Na > Mg > K > Ca > Fe. However, some differences are found in the order of major ions in the river water depending on sampling locations. Anions: SO_4_^2-^ > Cl^−^ > NO_3_^−^ > HCO_3_^−^ > PO_4_^3−^ > NO_2_^−^ > F^−^; Cations: SiO_2_ > Na > K > Ca > Mg > Fe. The high content of major ions in the effluent is due to the dominant anthropogenic pollutants from the industrial zone. According to the results of this investigation, effluents from the industrial sites could be ranked as strong wastewater, and a gradually deteriorating adjacent river body should not be discharged without prior treatment according to the standards.

### 3.2 Assessment of the water quality using EWQI, CCME WQI and DWQI

Physicochemical parameters were used to incorporate the water quality index (WQI) to generalize the condition of the water body based on the results integrated from different analyzed parameters. To calculate WQI methods, pH, EC, Ca, Mg, Na, K, HCO_3_, Cl, SO_4_, NO_3_ and F were considered for each sampling site's location, i.e., effluent sites and river water. Thirty water samples, including point sources (n = 10) and river water (n = 20) were used for the WQI calculation. Moreover, WHO and Bangladesh surface-water effluent discharge threshold values are referenced for the calculations. Three WQIs were chosen: the Entropy Water Quality Index (EWQI), the Water Quality Index (WQI), and the Canadian Council of Ministers of the Environment Water Quality Index (CCME WQI); as they are familiar and widely used to evaluate water quality [84,85].

**Fig. 2:**
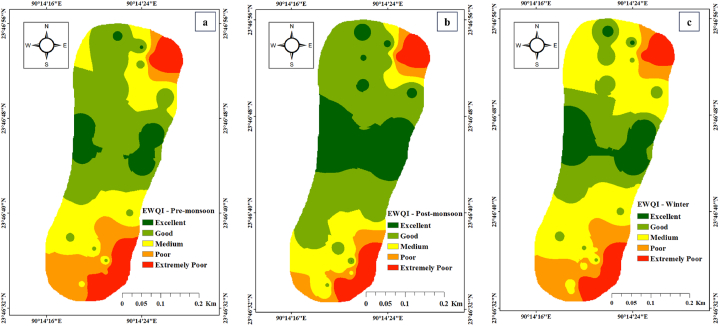

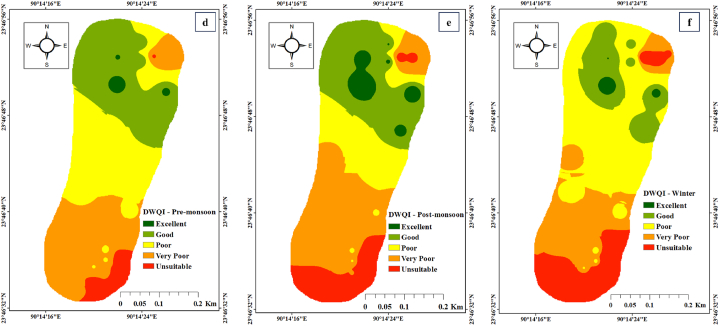
Spatial distribution for river water quality (a) EWQI – Pre-monsoon (b) EWQI – Post-monsoon (c) EWQI – Winter (d) DWQI – Pre-monsoon (e) DWQI – Post-monsoon and (f) DWQI – Winter.

Table 2 revealed that the effluent sites' water quality indices and river water depend on the criteria of EWQI, DWQI, and CCME methods. In this investigation, samples from effluent sites are characterized by extremely poor water quality. CCME WQI derived values of 8.83 in effluent sites and 40.74 in river water. The WQI and CCME WQI presented effluent sites with consistently worsened quality, i.e., ‘extremely poor’ (>150) and ‘poor’ (0–44) for the WQI and CCME, respectively; in the case of river water, the EWQI showed 75% ‘medium’, 5% ‘poor’ and 20% ‘extremely poor’ water quality (Table 2). WQI and CCME results varied due to different parameters, formulae, and reference standards [51,85,86]. Meanwhile, Fig. 2 illustrates the quality ratings of the indices derived from surface water samples. Effluent sites with the highest values revealed the presence of toxic constituents released from the industrial zone. This indicates that the sample quality may be thoroughly deteriorating. Equivalent trends were also observed in river water samples when considering effluent elements diluted by upstream flow in nearby areas. Fig. 2 illustrates the WQI and EWQI for the three-season sample from the tannery estate's zone of effect. The WQIs depicted in this picture were calculated using various modulation values to represent different contamination conditions. This study determined whether industrial wastewater was responsible for the area's poor water quality and the extent of contamination caused by effluent-derived heavy metals. This figure also shows the ability of heavy metals to contaminate surface water. To illustrate the overall capacity of heavy metals to impact water quality in this study area, we compared the EWQI and WQI based on seasonal variation. The findings imply that the tannery village is a possible source of HMs in river water.

Based on the study of the WQIs, treated effluent discharged from CETP containing harmful elements combined with river water should not be used for drinking unless a suitable technology exists to improve the surface water quality. Other industrial processes, as well as other anthropogenic sources, are likely contributing to the region's decreased water quality.

**Fig. 3:**
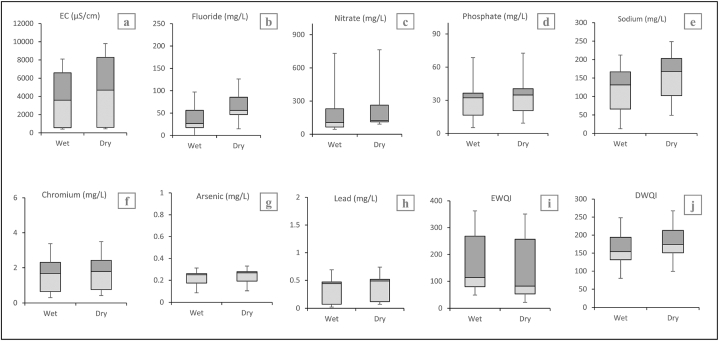
Illustration of seasonal variation by Box whisker plots of (a) EC, (b) Fluoride, (c) Nitrate, (d) Phosphate, (e) Sodium, (f) Chromium, (g) Arsenic, (h) Lead, (i) EWQI and (j) DWQI.

The ion distribution and HMs of the study area have been illustrated as shown in Fig. 3. This presents strong evidence that the Dhaleshwari River's flow is at its lowest throughout the winter and pre-monsoon season, and that seasonal fluctuations in the concentration of effluents released in its the watershed are negligible. Trace elements in surface water were highest in the winter and lowest after the monsoon. Except for Fe, Al, Zn, and Hg (Table 3) the range of average values for all other heavy metals exceeded the drinking standards' allowed limits recommended by WHO, USEPA, and DoE [23,70,96].

**Table 3:** Seasonal variation comparison of trace elements concentrations (mg/L) in Dhaleshwari river water with different permissible limits and other freshwater reservoirs around the world.

Metals	Observed Apr’ 21	Observed Oct’ 21	Observed Jan’ 22	Studies around the world			Standard for drinking water		
				**Iran** [93]	**Taiwan** [94]	**Turkey** [95]	**WHO** [96]	**USEPA** [70]	**DoE** [23]
Iron (mg/L)	0.341	0.264	0.190	–	–	0.018	0.3	0.2	0.3–1
Manganese (mg/L)	4.509	4.451	3.336	2.501	–	0.004	0.4	0.05	0.1
Nickel (mg/L)	1.732	1.057	1.108	1.104	0.030	0.002	0.07	0.02	0.1
Lead (mg/L)	0.456	0.343	0.390	–	0.051	0.000	0.01	0.01	0.05
Cobalt (mg/L)	0.602	0.458	0.475	–	–	0.000	0.1	–	0.1
Copper (mg/L)	0.614	1.506	1.561	0.122	0.105	0.001	2	2	1
Chromium (mg/L)	2.215	1.704	1.821	0.154	0.010	0.000	0.05	0.05	0.05
Cadmium (mg/L)	0.422	0.301	0.276	0.136	0.001	–	0.003	0.005	0.005
Mercury (mg/L)	0.015	0.014	0.016	–	0.002	–	0.006	–	–
Arsenic (mg/L)	0.184	0.179	0.164	0.172	0.003	0.003	0.01	0.01	0.05
Aluminium (mg/L)	0.297	0.240	0.223	–	–	0.023	–	–	–
Zinc (mg/L)	0.438	0.390	0.344	2.922	0.032	0.003	5	–	5

This finding emphasized the potential impact of tannery estate wastewater discharged near the research region. Two point source stations showed the highest levels of HMs (Fig. 2), signifying the effects of industries, manufacturing units, and CETP discharged pipelines. The midstream sites showed significantly low levels of ions due to their distant location from the tannery village.

### 3.3 Irrigation water quality assessment

The appraisal of surface water for irrigation purposes is a method for determining the adequacy of water for various applications. Water quality characteristics in the river are especially important during winter and pre-monsoon periods due to increased water demand, primarily for irrigation. Consequently, a water quality sampling design was better set up to analyze the water quality state during the summer season and verify its eligibility for irrigation applications. The feasibility of river water for irrigation was determined by computing Total Hardness (TH), Sodium Percentage (Na%), Sodium Adsorption Ratio (SAR), Soluble Sodium Percentage (SSP), Residual Sodium Bicarbonate (RSBC), Permeability Index (PI), Magnesium Adsorption Ratio (MAR), and Kelley's Ratio (KR). Table 5 presents the river water irrigation parameters for the pre-monsoon, post-monsoon, and winter seasons, respectively. Ca, Mg, K, Na, Cl, NO_3_, PO_4_, SO_4_, and HCO_3_ values in point source samples were beyond the permissible range; however, only a few river water samples in the post-monsoon period were within the acceptable range for irrigation water (Table 1).

**Table 4:** Classification of surface water quality based on presence of HMs of the study area.

Factor	Range	Rating	Suitability	% of Sample
Aluminum (mg/L)	Al < 5.0	3	High	100
	5.0 ≤ Al ≤ 20.0	2	Medium	0
	Al > 20.0	1	Low	0
Arsenic (mg/L)	As < 0.1	3	High	10
	0.1 ≤ As ≤ 2.0	2	Medium	27
	As > 2.0	1	Low	63
Cadmium (mg/L)	Cd < 0.01	3	High	0
	0.01 ≤ Cd ≤ 0.05	2	Medium	0
	Cd > 0.05	1	Low	100
Chromium (mg/L)	Cr < 0.1	3	High	0
	0.1 ≤ Cr ≤ 1.0	2	Medium	7
	Cr > 1.0	1	Low	93
Cobalt (mg/L)	Co < 0.05	3	High	0
	0.05 ≤ Co ≤ 5.0	2	Medium	100
	Co > 5.0	1	Low	0
Copper (mg/L)	Cu < 0.2	3	High	0
	0.2 ≤ Cu ≤ 5.0	2	Medium	100
	Cu > 5.0	1	Low	0
Fluoride (mg/L)	Fl < 1.0	3	High	0
	1.0 ≤ Fl ≤ 15.0	2	Medium	0
	Fl > 15.0	1	Low	100
Iron (mg/L)	Fe < 5.0	3	High	100
	5.0 ≤ Fe ≤ 20.0	2	Medium	0
	Fe > 20.0	1	Low	0
Lead (mg/L)	Pb < 5.0	3	High	100
	5.0 ≤ Pb ≤ 10.0	2	Medium	0
	Pb > 10.0	1	Low	0
Manganese (mg/L)	Mn < 0.2	3	High	0
	0.2 ≤ Mn ≤ 10.0	2	Medium	100
	Mn > 10.0	1	Low	0
Nickel (mg/L)	Ni < 0.2	3	High	0
	0.2 ≤ Ni ≤ 2.0	2	Medium	60
	Ni > 2.0	1	Low	40
Zinc (mg/L)	Zn < 2.0	3	High	100
	2.0 ≤ Zn ≤ 10.0	2	Medium	0
	Zn > 10.0	1	Low	0

**Table 5:** Classification of river water quality for irrigation purposes of the study area.

Parameters	Ranges	Class	Water Samples (%)		
			Pre monsoon	Post monsoon	Winter
EC	<700	Excellent	26.67	30	26
	700–3000	Good	13.33	10	14
	>3000	Fair	60	60	60
SAR	<10	Excellent	66.67	70	80
	10–18	Good	33.33	30	20
	18–26	Fair	0	0	0
	>26	Poor	0	0	0
RSBC	<5	Safe	60	60	60
	5–10	Marginal	26.67	27	23.33
	>10	Unsatisfactory	13.33	13	16.67
KR	<1	Excellent	0	0	0
	>1	Excess level of Na	100	100	100
MAR	<50	Excellent	43.33	60	6.67
	>50	Harmful effect to soil	56.67	40	93.33
TH	<75	Soft	100	100	100
	75–150	Moderately hard	0	0	0
	150–300	Hard	0	0	0
	>300	Very hard	0	0	0
Na%	<20	Excellent	0	0	0
	20–40	Good	0	10	0
	40–60	Permissible	3.33	23.33	16.67
	60–80	Doubtful	83.34	66.67	83.33
	>80	Unsuitable	3.33	0	0
SSP	<20	Excellent	0	0	0
	20–40	Good	0	0	0
	40–80	Fair	20	46.67	40
	>80	Poor	80	53.33	60

The average pH values were above 8.5 (Table 1), which is beyond the recommended safe range of 6.0–8.5 for irrigation water [87]. The average TDS level observed during the pre-monsoon was greater than 1000 mg/L, which is considered “doubtful to unsuitable” surface water [58]. The EC value in the examined samples was noted to determine different surface water parameters. The findings of this investigation show that the surface water EC values ranged from 524 to 9850 with a mean value of 4840, showing that most of the sampling region has good to fair quality for irrigation needs (Table 1 and 5). TH ranged widely between 1.20 and 36.93 meq/L, with an average of 6.43 meq/L (Table 5). All water samples were within the allowable limit (<75) and belonged to the category of soft water. The range of Na% values was from 24.21 to 78.68, with a mean of 61.02%. Most water samples were unfit for irrigation and fell within the doubtful range (60–80 Na%). Water having a Na% concentration of less than 40 meq/L is suitable for irrigation purposes. In the studied locations, tannery effluents in the Dhaleshwari River could cause elevated salinity. The SAR is commonly considered to be an efficient evaluation criterion for the majority of irrigated agricultural water. The magnitude of the Na–Ca/Mg exchange process between water and soil fine particles, which replaces adsorbed Mg and Ca with Na ions, is demonstrated by SAR. Soil hardness and reduced permeability result from this cation-exchange reaction. However, SAR values higher than 18 suggest a sodium hazard. The range of SAR values was 0.91–18, with an average of 6.12 (Table 5). SAR values less than 18 are good [57]. Furthermore, discernible seasonal variations in SAR values have been observed, including an increase during the winter and pre-monsoon. On the other hand, SSP (soluble sodium percentage) values ranged from 49.20 to 93.62 with a mean of 80.89, indicating fair to poor quality. Consequently, SSP showed (50–80%) water samples of poor quality and (20–40%) fair water quality for irrigation. The residual sodium bicarbonate (RSBC) values varied from 1.26 to 9.16 meq/L (Table 5), indicating that residual sodium carbonate is present in all water samples. These values are significantly higher than the 1.25 meq/L criterion for potable water and are consequently unsuitable for irrigation. However, the majority of water samples for PI from all seasons fall into classes I (>75) and II (25–75), indicating good irrigation quality. Kelly's ratio considers Na, Ca, and Mg contents for assessing surface water. Typically, surface water with Kelley's ratio below one is suitable for irrigation. All seasons with a KR greater than 1 imply that surface water is unsuitable for irrigation. Several water quality methods were used to evaluate irrigation water when the deirrigation rate for irrigation increased throughout the winter and before the monsoon season [88]. The suitability of surface water for irrigation depends on the ratios of EC and Na to other cations and anions. All of the examined parameters, i.e., RSBC, KR, MAR, Na%, and SSP, indicate that surface water is inappropriate for irrigation purposes.

### 3.4 Human health risk assessment

The objective of risk assessment is to minimize the health hazards imposed by potable water, which is attainable through several effective and practicable methods. Implementing control measures for a safe and sustainable water supply depends on monitoring toxic waste and polluted areas. The concentrations of metals present in river water and effluents were used to calculate the harmful effects of human exposure via the two separate routes of ingestion and dermal exposure.

#### 3.4.1 Non-carcinogenic risks

**Fig. 4:**
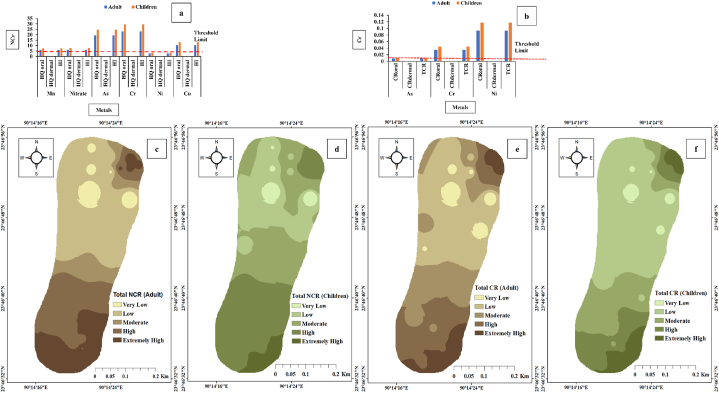
Diagram showing HQoral, HQdermal and HI (a) NCR and (b) CR; Geospatial distribution map of total (c) NCRAdult (d) NCRChildren (e) CRAdult and (f) CRChildren.

Humans are vulnerable to different contaminants via three routes: air intake, ingesting (drinking water), and directly to the skin [78]. The results of our investigation reveal the harmful conditions of the Dhaleshwari River due to certain heavy metals (HMs) at various sampling sites. Amount of heavy metals of water samples from Dhaleshwari River, Bangladesh are shown in Table 4. Non-carcinogenic adverse impacts on individuals from TEs are often assessed by determining Hazard Quotient (HQ) and Hazard Index (HI). The HQ and HI evaluated the non-carcinogenic risk for populations of adults and children based on several significant routes. HQ < 1 is considered acceptable, although values higher than unity imply a high likelihood of risk and are unacceptable. Dhaleshwari River water is a potential health hazard for people of all ages due to HQ > 1 values for several HMs at multiple sampling points. The values for HQ for different HMs are illustrated in Fig. 3. The detrimental health risks for adults and children have been observed at numerous sampling sites. The highest health hazard could be attributed to Cr ingestion with HQ > 1 at all the sampling points. Cr levels were found to be quite higher in the dry season, possibly as a result of seasonal changes in the river's water capacity, with lower water flow in the pre-monsoon season leading to the accumulation of the TEs in water, therefore increasing their concentration. Adults and children's HQ ranges for Cr were 8.56–29.38 and 10.87 to 37.44, respectively. The higher Cr absorption rate of children compared to adults exposed them to greater potential health hazards. The illnesses associated with adults' Cr consumption include gastritis, nephrotoxicity, and hepatotoxicity problems [89]. The health risk associated with these metals could be stated based on the findings from various sample locations as Cr > As > Cu > NO_3_>Mn > Ni > Zn > Pb (Fig. 4). The study river in northern Bangladesh, receives a substantial portion of As from highland Himalayan catchments. The HQ values for Cu and As are higher than unity at 60% of the sampling stations, thereby posing potential dangers to one's health from consumption. The variation of HQ for Pb for adults and children was 0.89–19.15 and 1.14 to 24.37, respectively. Diseases caused by Pb in adults include problems in the nervous system, reproductive system, nephrology, and cardiovascular system. This HM also affects the early phases of fetal growth in pregnant women [90]. The HQ range for As in adults and children at several sampling points was 4.99–28.75 and 6.36–36.60, respectively. Chronic oral intake of As may cause reproductive, neurological, and cardiovascular diseases [91]. Cu exposure has serious detrimental effects on the human lungs and kidneys. The other heavy metals (Fe, Mn, and Ni) do not pose any health risk, based on the value of HQ < 1 at all sampling stations for all three heavy metals.

HI in children and adults surpasses the permissible limit at all sampling points. Fig. 4 depicts the outcome of the HI. The graphical representation for HI (children and adults) adequately illustrates the variability at each sample site. Each sample point's value of HI is greater than unity, indicating the significant health risk that the numerous trace metals pose. The HI range for adults and children is 22.81–111.64 and 29.04–142.10, respectively. The maximum profound consequence for adults is 111.64 at the outlet source, indicating the degree of pollutants generated by HMs at the Central Effluent Treatment Plant (CETP) release point and the Tannery house. The highest risk score for children is also found at the exact sampling location. It is 142.10, indicating the likelihood of HMs contamination in water released from the CETP after treatment. The slightest health risk is observed at the mid-river due to its position roughly 500 m distant from outlet drainage, reducing the probability of tannery effluent effects.

#### 3.4.2 Carcinogenic risks

The quantity of toxic metals found in the water body may be calculated by measuring the concentration of trace elements. Only As, Pb, Ni, and Cd are considered when figuring out the CR index since their carcinogenic slope factors (CRFs) are known. The range of CR for the ingestion route was 0.0427–0.2912 (mean 0.1361) for adults and 0.0544 to 0.3706 (mean 0.1732) for children. The computed risk of cancer, related to HMs ingestion indicated that all of the water samples exceeded the maximum acceptable level recommended by the USEPA (i.e., 1 × 10^−4^) indicating the potential for major health effects for the local population (Fig. 4). Ni and Cr were the most common contributors to the TCR (CRingestion + CRdermal) via both the ingestion and dermal contact pathways. Due to increasing pollution status, fast industrialization, and other economic activity, HQ and HI pollution may surpass the risk limit, endangering nearby inhabitants.

## 4 Conclusions

Major rivers in Bangladesh close to urbanized and industrialized areas lose their purity due to contamination. The effluents of the industries, even after being treated for a certain aggregate, are a major environmental, health, and social issue. Surface water is still the primary source of irrigation and anthropogenic purposes in the Dhaleshwari river zone. This study gathered samples to observe the degree of contamination based on seasonal fluctuations. Yet, the investigation results show that treated effluents from CETP had extremely high values and ultimately found their way into the river, causing alarming water quality. The values of the parameters pH, EC, Eh, DO, TDS, salinity, anions, cations, and metal (oid)s exceeded the threshold values prescribed by the DoE for effluent discharge in surface water and the WHO for drinking purposes. However, a different WQI method was used to evaluate the water quality in the adjacent river of Savar tannery village. The results showed that most parameters varied significantly among the effluent and river water sampling points. The water quality was generally “extremely poor” in the point sources and “moderate” in the mid-river according to the index classification during our study period. Based on spatial distribution maps, the WQIs of the vicinity river show a decrease in toxic concentration with distance, even though the water is not suitable for consumption. TH, Na%, SAR, SSP, RSBC, PI, MAR, and KR were assessed for surface water quality assessment in the area of study for irrigation purposes. The data evaluation revealed that most sample water for irrigation was “unsuitable.” Results concluded from this study reveal a high amount of HMs presence in the river Dhaleshwari. The heavy metal concentrations in overall water quality are followed in decreasing order: Mn > Ni > Cr > Co > Cd > Pb > Zn > Al > As > Hg. In addition, the health hazard indicated the considerable health impacts related to water intake at each sampling location. The ranges of HQ transcend unity and raise serious concerns regarding the potential exposure of adults and children to danger. HI has increased at every sampling location for adults and children, indicating the possible health risk posed by numerous HMs. In most samples, the carcinogenic risks were found to be greater for children than adults. Contaminations have spread out in such proportion that they have been visually observed in the nearby river scenery. The result, in consequence, tends to recommend the authority take a proper, effective maintenance approach to CETP and areas in need of remediation to maximize net benefits through reducing pollution loads. Despite the aforementioned investigation, there are also some drawbacks to the study that might be addressed in further research. The current research did not consider wastewater integrity before and after CETP processing due to a lack of incorporation authority. The sheer quantity of sampling locations for river water is likewise limited. Therefore, it is recommended that additional studies broaden their scope to include more Dhaleshwari river water stations in order to conduct more comprehensive, encompassing, and comparative assessments. Finally, the use of river water for consumption, i.e., drinking and irrigation purposes, exposes the population to health risks. This study implies that the current condition of the surface water may act as a model to find out the potential risk to human health soon.

### 4.1 Recommendation for sustainable river water protection and management

The development of a tannery village on the Dhaleshwari riverfront was a concerning issue for the vicinity of Hemayetpur, Savar. The inadequacy of execution concerning minimum ecological disturbance must have been reevaluated and improved. The maintenance of CETP on the riverside must be evaluated and rectified.

• Excessive disposal of industrial effluent is terminated immediately, and it is necessary to implement a strategy for handling wastewater.
• It is necessary to increase the capacity of existing Water Treatment Plants (WTPs) proportionally concerning the pace of wastewater generation due to the growing tannery houses. The maintenance and frequent surveillance of WTPs to ensure their efficient functioning and competence are crucial.
• Implementing “Green projects” along the riparian zone as part of the riverfront tannery village may prove effective in achieving “Leed Certification”. It is necessary to implement methods for managing CETP feasibly.
• Adding more water to the river to improve water quality can be remarkably effective.
• Lake, pond, and river regeneration can generate water for the rejuvenation of the Dhaleshwari river.
• Solid waste segregation is necessary to prevent metal intrusion into the river by eliminating illicit disposal along the river channel.

## Ethical approval and consent to participate

This research does not involve human trials and the interviewees understand the research concept and have complete authorization, thus ethical approval is not required. All interviewees authorized interview information disclosure.

## Consent for publication

All presentations of this study have consent for publication.

## Author contribution statement

Hazzaz Bin Hassan: Performed the experiments; analyzed and interpreted the data; analysis tools or data; wrote the paper.

Md. Moniruzzaman: Conceived and designed the experiments; contributed reagents, materials, analysis, and interpreted the data.

Ratan Kumar Majumder: Contributed reagents, materials, analysis and interpreted the data.

Fowzia Ahmed: Analysis tools or data, analyzed and interpreted the data.

Md. Abdul Quaiyum Bhuyian, and Md Ariful Ahsan: Performed the experiments, contributed reagents, materials, analysis data.

Hafiz Al- Asad: Performed the experiments, analyzed and interpreted the data.

## Data availability statement

Data will be made available on request.

## Declaration of competing interest

The authors declare that they have no known competing financial interests or personal relationships that could have appeared to influence the work reported in this paper.
